# Thermal equilibrium as a predictor of growth efficiency in preterm infants

**DOI:** 10.3389/fped.2024.1469724

**Published:** 2024-11-05

**Authors:** Julia Heiter, Juliane Konow, Jochim Koch, Dominique Singer, Chinedu Ulrich Ebenebe

**Affiliations:** ^1^ Division of Neonatology and Pediatric Critical Care Medicine, University Medical Center Hamburg-Eppendorf, Hamburg, Germany; ^2^ Children’s Hospital, Itzehoe, Germany; ^3^ Formerly Research Unit, Draegerwerk AG & Co. KGaA, Luebeck, Germany

**Keywords:** preterm infants, growth rate, caloric intake, heat exchange, calculation program, incubator settings

## Abstract

**Introduction:**

Providing adequate nutrition to preterm infants to achieve postnatal growth similar to intrauterine growth remains challenging due to the unpredictability of individual determinants.

**Material and methods:**

We used a calculation program for infant incubators to compare the estimated heat balance with the caloric intake and growth rate in Very Low Birth Weight Infants (VLBWI).

**Results and discussion:**

A group of 32 VLBWI was studied over a period of 14–28 days. An interrelationship between thermal equilibrium and growth rate was observed, with standardized incubator settings being unable to avoid periods of negative thermal balance and concomitantly poor growth rate.

**Conclusion:**

Determining personalized incubator settings by means of a calculation program could help improve nutrition and growth in preterm infants.

## Introduction

One of the goals of nutritional practices for preterm infants is to provide an adequate energy supply to achieve growth rates like those of fetuses *in utero* (1–4). Basically, growth occurs when caloric intake exceeds energy expenditure. However, determining the energy expenditure in very low birth weight infants (VLBWI) remains challenging, given the technical obstacles to the use of direct or indirect calorimetry during incubator care (5–7).

The environment to which preterm infants are exposed after birth differs considerably from the maternal uterus, particularly in terms of heat exchange (7–10). After birth, there is an increase in basal metabolic rate within one to two days in term babies and two to three weeks in VLBWI (10–12), adapting endogenous heat production to their large surface-to-volume ratio. In addition, ambient temperature fluctuations are increasingly compensated by the onset of thermogenesis in brown adipose tissue (9, 10).

The postnatal changes are reflected in the incubator settings which, starting from high air temperature and relative humidity, can be gradually lowered. The more the preterm baby contributes to its thermal stability, the less the ambient values need to be kept high. In this respect, an intensive care incubator behaves like a (compensatory) “calorimeter”.

This study attempted to utilize a calculation program for incubator settings to determine the thermal equilibrium of preterm infants as a potential predictor of growth efficiency.

## Material and methods

### Study design and subjects

This was a prospective observational study at the tertiary perinatal center of the University Medical Center Hamburg-Eppendorf over a 12-month period (May 2019–2020). Inclusion criteria were preterm neonates with a birth weight < 1,800 g and a gestational age < 33 weeks, who were admitted to the Neonatal Intensive Care Unit (NICU) and treated in an incubator (Caleo®, Draeger, Luebeck, Germany) for at least 14 days. Patients with chromosomal aberrations, congenital malformations, small for gestational age (birth weight < 3rd percentile), and with surgical interventions were excluded from the study. Neonates were eligible for enrollment if parental consent was obtained.

Parenteral nutrition with glucose was started immediately after birth. Electrolytes, amino acids, and lipids were added within the first three days of life. Enteral nutrition with increasing amounts of breast milk or preterm formula was also initiated within the first three days of life. The incubator settings were selected by the nursing staff in accordance with the ward's internal practices (Table 1) to keep the preterm infants’ rectal temperature in the normothermic range (36.5–37.5°C).

**Table 1:** Unit-specific incubator settings for temperature and humidity that were used for the preterm infants in this study.

Birth weight	Day of life/temperature settings	Gestational age	Week of life/humidity settings
<1,000 g	1 37°C + 2–3 Skin mode 4–7 Skin mode 8− Skin mode	<28 weeks	1 75% 2− Reduction by 5% per week
<1,500 g	1 36–34°C 2–3 35–33°C 4–7 34–33°C 8− 33–32°C	28–<30 weeks	165%–60% 2− Reduction by 5% per week
<2,000 g	1 34–33°C 2–3 33°C 4–7 33–32°C 8− 32°C	30–<33 weeks	1 50% 2− 50%

Relevant clinical patient data, daily energy intake, and the incubator settings chosen by the caregivers were extracted from the digital patient data management system (ICM®, Draeger, Luebeck, Germany) on days 1, 2, 3, 4, 5, 6, 7, 10, 14, 18, 22, and 28 of life. The thermal equilibrium was assessed using the HeatBalance® program (Draeger Medical Germany, Luebeck, Germany) (13, 14), originally developed to simulate appropriate incubator settings based on physical algorithms. As the calculation was carried out retrospectively and without feedback to the nursing staff, any influence on the caregivers’ actual settings could be ruled out.

**Figure 1:**
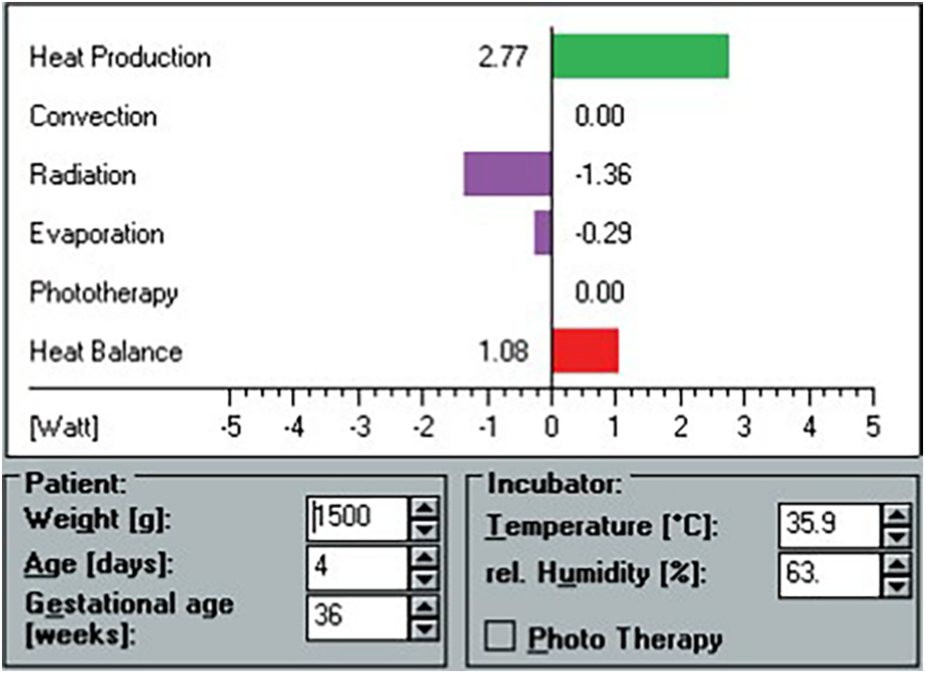
Screen view example of the HeatBalance® program. Heat production, heat losses, and heat balance are displayed after entry of patient and incubator data.

The HeatBalance® program accounted for the following parameters: the baby's heat production (Q_neonate_, W), the (negative or positive) heat fluxes due to convection (Q_conv_, W), radiation (Q_rad_, W), evaporation (Q_evap_, W), respiration (Q_resp_, W), and phototherapy (Q_pt_, W), as well as the resulting water loss (ml/kg/h). Heat production was estimated based on the baby's body weight, corrected gestational age, and activity level based on data from the literature (8, 13, 14). Total heat loss (Q_total_, W) was defined as the sum of all heat fluxes (Q_total_ = Q_conv +_ Q_rad +_ Q_evap +_ Q_resp +_ Q_pt_). The heat balance was the difference between heat production and total heat loss (Q_hb_ = Q_neonate—_Q_total)_. Required independent variables for the program's algorithms included weight (g), gestational age (weeks), postnatal age (days), room temperature (°C), incubator air temperature (°C), incubator relative humidity (%), ventilation (yes/no), phototherapy (yes/no), and clothing (yes/no) (Figure 1). For reasons of comparability with clinical habits, values expressed in watts by the HeatBalance® program were later transformed into calories per unit of time (1 W = 860 cal/h).

The study protocol was approved by the ethics committee of the local Medical Chamber of Hamburg (2020-10105-BO-ff).

### Statistics

Statistical analyses were performed using SPPS Ver. 27 (IBM, NY, USA) and SigmaPlot Ver. 14 (Systat Software, Inc., CA, USA). Data on neonatal demographics were expressed as median and range for continuous variables. Mean values and standard deviations (SD) of caloric intake, absolute and specific weight gain, heat production, and heat balance were plotted against the newborn's postnatal age.

## Results

### Demographic data and body weight

The eligible study population consisted of 32 preterm neonates with a median (range) gestational age and birth weight of 29.1 (25.6–32.9) weeks and 1,213 (720–1,795) g, respectively. Nine consecutive measurements (days 1–14) were carried out in twelve patients, and twelve data points (days 1–28) were recorded in 20 patients. Incubator settings were made according to our institutional regimen, with humidities between 50% and 75% for one week, depending on gestational age, followed by a 5% reduction per week. Temperature settings started between 34 and 37°C depending on birth weight and were regulated by servo (skin control) mode (Table 1).

**Figure 2:**
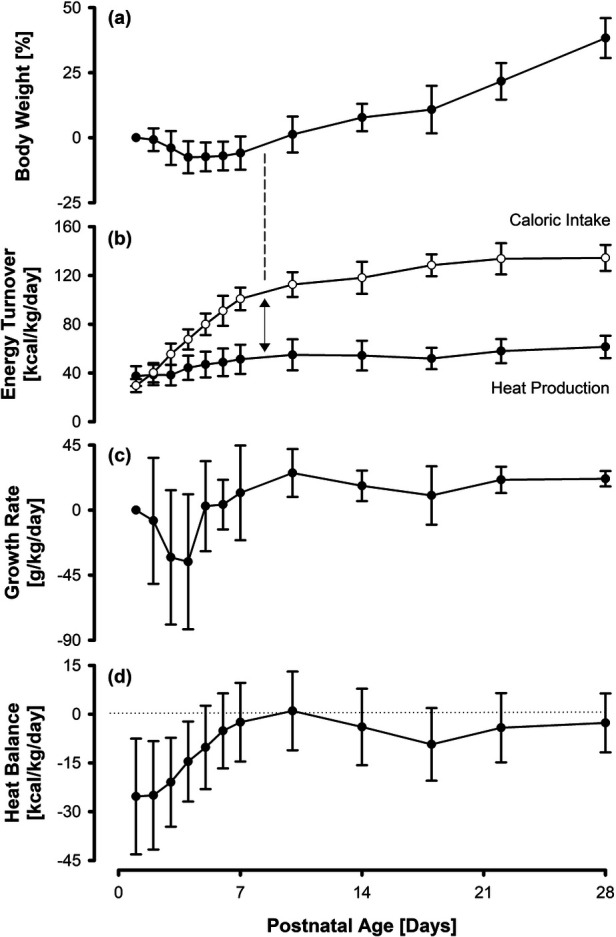
Percentage body weight **(a)**, nutritional caloric intake and estimated heat production **(b)**, specific growth rate **(c)**, and net heat balance **(d)** in 32 VLBWI. Growth sets in as soon as an energy excess (caloric intake minus heat production) of around 50 kcal/kg/d is achieved/exceeded. Overall, the specific growth rate parallels the net heat balance, with a negative heat balance being accompanied by a temporary retardation in growth rate.

Body weight initially dropped to a mean (SD) minimum of −7.5 (±6.2)% on day four, followed by a rise to 38.3 (±7.7)% above birth weight on day 28 (Figure 2a).

### Caloric intake and heat production

Consistent with our institutional nutritional regimen, daily caloric intake per weight increased steadily up to a maximum mean of 137 (±9) kcal/kg/d on day 28 of life. The mean heat production estimate was 37.5 (±8.2) kcal/kg/d directly after birth and increased to 61.5 (±9.2) kcal/kg/d on day 28. The mean difference between caloric intake and heat production (energy excess) increased from −7.8 (±9.8) kcal/kg/d on the first day of life to 73.0 (±16.4) kcal/kg/d on day 28. The increase in body weight started once the caloric intake exceeded the heat production rate by roughly 50 kcal/kg/d (Figure 2b).

### Growth rate and heat balance

The growth rate varied to a maximum of 25.7 (±16.6) g/kg/d. The heat balance started at −25.3 (±17.8) kcal/kg/d after birth and reached a temporary steady state by day 10. However, within the following 8 days, it dropped again to a minimum of −9.3 (±11.2) kcal/kg/d, stabilizing at −4.2 (±10.6) kcal/kg/d from day 22 onwards. The variations in growth rate appeared to run parallel to the fluctuations in heat balance (Figures 2c,d).

## Discussion

It has long been known that thermal care affects the mortality and morbidity of newborn infants (8). Appropriate incubator settings are usually selected empirically using textbook recommendations or in-house standard operating procedures (cf. Table 1) (10, 15). However, reaching the target body temperature (mostly ±37°C) does not prevent preterm babies from expending extra energy to compensate for a residual thermal imbalance. In fact, by using a simulation program for incubator settings, a partly negative heat balance of VLBWI on our NICU was detected, which appeared to affect growth rate apart from caloric intake.

The 7.5% initial weight loss in the first week of life mainly reflects the disruption of nutrient supply and the shift in body water content related to preterm birth (16). However, even slightly suboptimal incubator conditions (not recognizable by the preterm infants’ rectal temperatures) may lead to avoidable water loss and additional heat production (beyond the basal rate estimated by the calculation program).

The final weight gain of approximately 20 g/kg/d, meeting international guidelines (1–4), was achieved in the fourth week of life when the caloric intake (135 kcal/kg/d) exceeded the estimated heat production rate by roughly 75 kcal/kg/d. Therefore, infants required a caloric intake of approximately 6–7 (corresponding to an energy excess of 3–4) kcal per gram weight gain. This is slightly higher than older data on the energetic cost of growth in preterm infants, who were less immature and grew more slowly at the time (17, 18).

The variations in specific weight gain, paralleling the—albeit slight—thermal imbalances occurring in the second and third weeks of life, reveal that there is still room for improvement in the thermal care of VLBWI. Similar to recent nutritional concepts promoting optimization of daily caloric intake based on individual growth trajectories (19, 20), individual adjustment of incubator settings using a calculation program could optimize the growth efficiency of preterm neonates.

This study has some limitations. First, the caloric intake was estimated based on tables showing the average energy content of breast milk and infant formula. However, as the caloric density of breast milk can vary widely, the actual individual intake may differ considerably from the one assumed. Second, the parameters used in the HeatBalance® program are also based on stored table values (8, 13, 14). This is particularly true for the heat production (resting metabolic rate) which, if measured directly, would allow a more accurate determination of the babies’ caloric needs. Third, this retrospective chart evaluation only provides indirect evidence for the potential benefit of personalized vs. table-based incubator settings and needs to be confirmed by a prospective study.

In conclusion, however, the present data suggest that a more individual adjustment of thermal environmental conditions could be an effective and comparatively simple way of further optimizing growth efficiency in preterm infants.

## Data Availability

The raw data supporting the conclusions of this article will be made available by the authors, without undue reservation.

## References

[1] Agostoni C Buonocore G Carnielli VP De CM Darmaun D Decsi T. Enteral nutrient supply for preterm infants. A comment of the ESPGHAN committee on nutrition. J Pediatr Gastroenterol Nutr. (2010) 50:85–91. 10.1097/MPG.0b013e3181adaee0 19881390

[2] World Health Organization (ed). WHO recommendations for Care of the Preterm or Low-Birth-Weight Infant. Geneva: World Health Organization (2022). Available online at: www.who.int/publications/i/item/9789240058262 (visited September 25, 2024). 36449655

[3] Young A Beattie RM Johnson MJ. Optimising growth in very preterm infants: reviewing the evidence. Arch Dis Child Fetal Neonatal Ed. (2023) 108:2–9. 10.1136/archdischild-2021-322892 35228320

[4] Meiliana M Alexander T Bloomfield FH Cormack BE Harding JE Walsh O Nutrition guidelines for preterm infants: a systematic review. JPEN J Parenter Enteral Nutr. (2024) 48:11–26. 10.1002/jpen.2568 37855274

[5] Sauer PJJ Visser HKA. Calorimetry of newborn infants: techniques and applications. Thermochim Acta. (1991) 193:49–56. 10.1016/0040-6031(91)80173-G

[6] Bauer K Pasel K Uhrig C Sperling P Versmold H. Comparison of face mask, head hood, and canopy for breath sampling in flow-through indirect calorimetry to measure oxygen consumption and carbon dioxide production of preterm infants <1500 grams. Pediatr Res. (1997) 41:139–44. 10.1203/00006450-199701000-00022 8979303

[7] Singer D. Thermometry and calorimetry in the neonate: recent advances in monitoring and research. Thermochim Acta. (1998) 309:39–47. 10.1016/S0040-6031(97)00427-9

[8] Okken A Koch J (ed). Thermoregulation of Sick and low Birth Weight Neonates: Temperature Control, Temperature Monitoring, Thermal Environment. Berlin: Springer (1995).

[9] Agren J. The thermal environment of the intensive care nursery. In: Martin RJ Fanaroff AA Walsh MC, editors. Fanaroff and Martin’s Neonatal-Perinatal Medicine: Diseases of the Fetus and Infant, 10th ed. Philadelphia: Elsevier Saunders (2015). p. 502–12. Chapt 36.

[10] Singer D van der Meer F Perez A. What is the right temperature for a neonate? Pediatr Adolesc Med. (2020) 22:95–111. 10.1159/000495437

[11] Bauer K Laurenz M Ketteler J Versmold H. Longitudinal study of energy expenditure in preterm neonates &lt;30 weeks’ gestation during the first three postnatal weeks. J Pediatr. (2003) 142:390–6. 10.1067/mpd.2003.143 12712056

[12] Bauer J Werner C Gerss J. Metabolic rate analysis of healthy preterm and full-term infants during the first weeks of life. Am J Clin Nutr. (2009) 90:1517–24. 10.3945/ajcn.2009.28304 19812174

[13] Wasner J. Heat Balance of Premature Infants. Lübeck: Draegerwerk AG (1994).

[14] Lyon A Püschner P. ThermoMonitoring—a Step Forward in Neonatal Intensive Care. Lübeck: Draegerwerk AG (2015).

[15] Perez A van der Meer F Singer D. Target body temperature in very low birth weight infants: clinical consensus in place of scientific evidence. Front Pediatr. (2019) 7:227. 10.3389/fped.2019.00227 31231623 PMC6568209

[16] Segar JL. A physiological approach to fluid and electrolyte management of the preterm infant: review. J Neonatal Perinatal Med. (2020) 13:11–9. 10.3233/NPM-190309 31594261

[17] Reichman BL Chessex P Putet G Verellen GJ Smith JM Heim T Partition of energy metabolism and energy cost of growth in the very low-birth-weight infant. Pediatrics. (1982) 69:446–51. 10.1542/peds.69.4.446 7070891

[18] Micheli JL Pfister R Junod S Laubscher B Tolsa JF Schutz Y Water, energy and early postnatal growth in preterm infants. Acta Paediatr Suppl. (1994) 405:35–42. 10.1111/j.1651-2227.1994.tb13396.x 7734789

[19] Rochow N Fusch G Mühlinghaus A Niesytto C Straube S Utzig N A nutritional program to improve outcome of very low birth weight infants. Clin Nutr. (2012) 31:124–31. 10.1016/j.clnu.2011.07.004 21890250

[20] Landau-Crangle E Rochow N Fenton TR Liu K Ali A So HY Individualized postnatal growth trajectories for preterm infants. JPEN J Parenter Enteral Nutr. (2018) 42:1084–92. 10.1002/jpen.1138 29419902

